# A Fully Sensorized Cooperative Robotic System for Surgical Interventions

**DOI:** 10.3390/s120709423

**Published:** 2012-07-09

**Authors:** Saúl Tovar-Arriaga, José Emilio Vargas, Juan M. Ramos, Marco A. Aceves, Efren Gorrostieta, Willi A. Kalender

**Affiliations:** 1 Institute of Medical Physics, Friedrich-Alexander-University Erlangen-Nuremberg, Henkestr. 91, 91052 Erlangen, Germany; E-Mail: willi.kalender@imp.uni-erlangen.de; 2 Informatics Faculty, Autonomous University of Querétaro, Avenida de las ciencias s/n, Juriquilla, Querétaro, Qro. C.P. 76230, Mexico; E-Mails: emilio@mecatronica.net (J.E.V.); jramos@mecamex.net (J.M.R.); marco.aceves@uaq.mx (M.A.A.); efrengorrostieta@gmail.com (E.G.)

**Keywords:** surgical robotics, robotic needle-placement, robot-driven C-arm, light-weight robot

## Abstract

In this research a fully sensorized cooperative robot system for manipulation of needles is presented. The setup consists of a DLR/KUKA Light Weight Robot III especially designed for safe human/robot interaction, a FD-CT robot-driven angiographic C-arm system, and a navigation camera. Also, new control strategies for robot manipulation in the clinical environment are introduced. A method for fast calibration of the involved components and the preliminary accuracy tests of the whole possible errors chain are presented. Calibration of the robot with the navigation system has a residual error of 0.81 mm (rms) with a standard deviation of ±0.41 mm. The accuracy of the robotic system while targeting fixed points at different positions within the workspace is of 1.2 mm (rms) with a standard deviation of ±0.4 mm. After calibration, and due to close loop control, the absolute positioning accuracy was reduced to the navigation camera accuracy which is of 0.35 mm (rms). The implemented control allows the robot to compensate for small patient movements.

## 1. Introduction

Surgical robotics is an evolving field with a relatively short history. The first recorded medical application occurred in 1985, where a brain biopsy was carried out [1]. Surgical robotics is an interdisciplinary field in which many components interact with each other. These include electromechanical devices such as motors, gears and a variety of sensors. Surgical robots have a lot of potential to improve patient care [2]. They have certain advantages over humans, for example, they don’t have the 20 Hz tremor (inherent to humans) and can follow smooth trajectories with more accuracy. In operations, where the physicians are close to radiation, the use of a robotics system can help to avoid exposure.

By definition, a surgeon is a hand worker. He uses his hands to cut tissues with scalpels and scissors, employ handsaws to cut bones, introduce screws, sew with thread and wire, *etc.* In order to execute these activities with a robot system, it has to be especially well equipped with vast range of internal and external sensors. Sensor technology is very important in modern operating rooms and will be essential in the operating rooms of the future [2]. Surgical robotics systems are possible due to the use of a wide variety of sensors. In contrast to robotics deployed in the automation industry, where robot assemblies are isolated from humans, a surgical robot exerts forces directly on the patient organs [3]. Designing a robot that touches, presses and cuts directly the both fragile and vital organs presents a number of issues. Those issues have made to slow down the practice of this very promising field.

In spite of all the challenges having to do with surgical robotics there are some successful systems that have FDA acceptance and are commercial available [4,5]. For instance, the Da-Vinci robot (Intuitive Surgical Inc., Sunnyvale, CA, USA) [4] is the system with more market penetration, as already more than 1933 units have been sold around the World. Its design gives surgeons increased dexterity while they work through small incisions in the body. The system includes a cockpit where the surgeon teleoperates the robot by using haptic devices, a cart with four arms (three of them, depending of the task, may have tweezers, scissors, hold a scalpel, and the other arm holds a laparoscope) and image processing equipment. Another successful surgical robot is the CyberKnife^®^ (Accuray Inc., Sunnyvale, CA, USA) [5]. The system has the ability to irradiate tumors very precisely even while the patient anatomy is moving due to breathing [6]. The main idea of this approach is to avoid damaging the healthy tissue around the tumor. The system comprises a linear accelerator mounted on the wrist of six degrees of freedom robot arm. The robot system task is to precisely orientate the linear accelerator towards the tumor. For vision, the system uses two orthogonal x-rays cameras equipped with flat panel detectors. For better accuracy, the system includes a navigation system for real-time patient tracking. Both the Da Vinci and CyberKnife systems make use of a variety of sensors necessary to perform their tasks.

Analyzing some successful surgical robotic systems, it is possible to figure out that they are isolated efforts using different technology levels. Experts realize that in spite of many offered solutions trying to introduce automation in the clinical environment, it is still too far away from the standardization level achieved by industry. Standardization is the key of surgical robotics development [2].

The mechanical part of a robot uses special sensors to measure the position not only of the end effector but also along its kinematics. The most used technologies for measuring position and its derivatives are resolvers, optical encoders and magnetic encoders with hall sensors. The sensing technology has opened the door to the development of robotic systems that are able to interact with changing environments. Hirzinger *et al.* (Institute of Robotics and Mechatronics of German Aerospace Center) developed a robot system for safe interaction with humans [7]. The robust compliance control of these systems allows the user to pull or push the robot arm with the hand and the system will move as if it had no weigh. This way of control is normally referred as “soft robotics” or “hands-on robots”. The MiroSurge system, which uses similar control schemes, was designed for minimally invasive surgical operations [8].

The main advantage of robotic assistance is the possibility to enhance or extend the hands and eyes of the surgeon during surgery. Controlling a surgical robotic system must be easy and intuitive for users that are not familiarized with robotics. Therefore, in the development of a surgical robot, a proper human-machine interface is essential. The word haptics is defined in robotics as the real and simulated touch interactions between robots, human and real, remote, or simulated environments [9]. The Da-Vinci system, for example, utilizes a haptic device for the intuitive manipulation of the robot [4]. Other surgical systems use joystick [10] and haptic devices like the Phantom Omni device (Sensable Technologies Inc., Wilmington, MA, USA).

One essential part in the development of surgical robotics is its capability to visualize the operating area. It is useful to classify visualization sensing modalities in real-time methods, which provide a continuous visualization of the area of interest, and non-real-time methods that are typically use for preoperative diagnosis and planning [11].

Commonly used real-time sensing modalities are endoscopes, ultrasound, fluoroscopy and optical coherence tomography (OCT). Endoscopes have been the most successful way of visualization. They are usually used for minimally invasive operations [5,12]. Unfortunately, they cannot provide further information from inside the tissue. In contrast, ultrasound provides 2D-real-time pictures from inside the tissue but only a skilled clinician is able to use this technique properly. Fluoroscopy is a technique that offers high spatial resolution so that submillimeter-sized objects can be resolved. Its 2D image clearly shows contrast between different materials (such as bone and liver) and different tissue densities (such as the heart and lungs) [13]. The biggest limitation of fluoroscopy is that overlaying structures are all reduced to a single image plane.

Most important non-real-time visualization systems deployed in surgical robotics are X-rays, Computed Tomography (CT), Magnetic Resonance (MR) and Positron Emission Tomography (PET). Previous work has been done using CT, (e.g., the ROBODOC and CASPAR system [14,15]) used for total hip and knee replacement. X-ray technology is employed in the CyberKnife system for localizing the target position. MR is the imaging technique that offers the most accurate tissue differentiation. Recently, specially designed robots, built out of non-metal parts, can be used together with MR [16].

FD-CT is a technology which combines fluoroscopy (real-time) and CT (non-real-time) in one single device, comprised of a C-arm equipped with flat panel detectors. FD technology in comparison to X-ray film and image intensifiers offers higher dynamic range, dose reduction, fast digital readout, yet keeping to a compact design [17]. Although FD-CT provides higher spatial resolution than common CT, it encompasses a few disadvantages, such as smaller field of view and lower temporal resolution [17]. Nevertheless, FD-CT has already proved unique for planning and intraoperative surgery [18–20]. C-arms are characterized by their flexibility and ease of use; in particular, by the possibility of choosing arbitrary angulations.

One of the impediments of using robot systems with imaging devices is that the gantry size of the later is not big enough to house both the patient and kinematics of some robots [21]. Many collision issues arise from these kind setups and this is one of the reasons that many researchers have built small dedicated robot systems which fit into the remaining place. A newly FD-CT system (Artis zeego, Siemens Healthcare, Forchheim, Germany) employs a robot arm (KUKA Robots, Gersthofen, Germany) for increased movement flexibility [17,22]. This system can be used for flexible intraoperative imaging and could be coordinated with other robotic systems to assist the surgeon.

Besides encoders and imaging devices as sensing modalities, localizers have been studied in order to analyze its benefits for surgical robotics applications [23,24]. These devices track the position of instruments relative to the patient anatomy. The instrument could be a surgical tool held by a robotic arm. In the CyberKnife system, an optical localizer is deployed to track the position of the patient prone to movements due to respiration [5].

In this work, the concept and implementation of a fully sensorized robotic surgical system is presented. The proposed system utilizes a variety of concepts employed in surgical robotics such as haptics, soft robotics, visualization and external tracking. The surgical system is comprised of two coordinated robot arms. The former carries out the surgical task and the second gives precise target visualization. The system was adapted for threating injuries where the insertion of a needle into an anatomy is commonly carried out in order to extract tissue samples for further analysis or to inject substances for therapy. The authors emphasize the description of the sensor technology employed in the system. In addition to conventional standards normally employed in the medical environment, the system utilizes standards from the automation industry.

## 2. Materials and Methods

**Figure 1.**
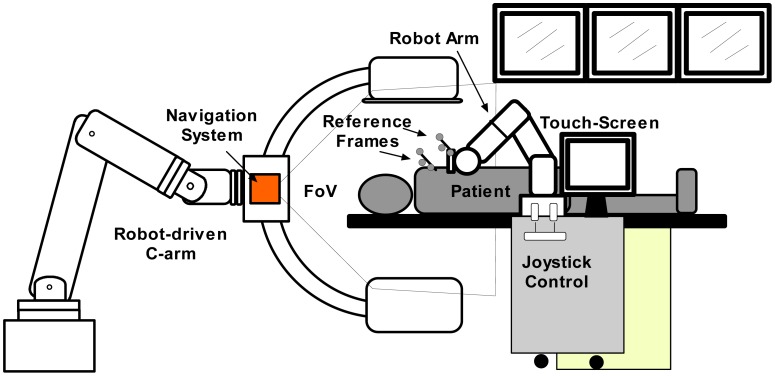
Sketch of a fully integrated system for percutaneous procedures.

In Figure 1, a representation of the main system components is displayed. For needle insertion, a serial robotic system is utilized. It has a special needle holder attached to its wrist. This mechanism allows the clinician to insert the needle manually. The robotic system comprises of a real-time controller (from the manufacturer) and an application controller. It was designed to be manually controlled by means of a touch screen and an industrial joystick. In order to position the robotic system easily along the CT table all the components are built on a mobile trolley. In this way, the system can be easily positioned and removed from the patient table. For target visualization, a robot-driven angiography system, equipped with a flat-panel detector, is deployed. This special C-arm can be positioned along the operating table providing full body coverage. Contrary to conventional C-arms, which only rotate around a fixed position, this imaging system can be adjusted to scan anatomical targets with different angles and convex shaped trajectories. Once a scan is taken, it sends the reconstructed 3D-images to the application controller for planning. In addition, 2D projections can be used to get a real-time target visualization. Based on the images, the surgeon can choose a target and an appropriate entry point. The other important part of the system is an optical localizer which tracks with precision the needle position by means of the reference frame that is attached to the needle holder. Additionally, it also tracks the patient position by means of a reference frame attached to it.

**Figure 2.**
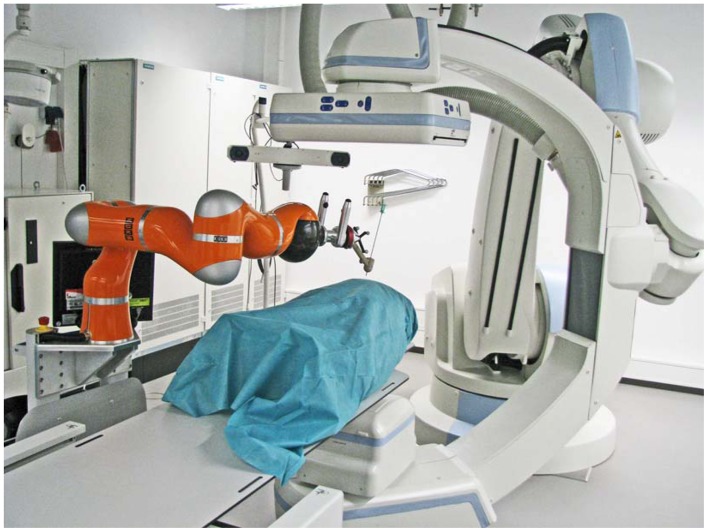
System setup in the interventional suite.

The natural haptic feedback while inserting a needle gives important information from the characteristics of surrounding tissue through the trajectory. Among these characteristics it is possible to get a feedback feeling of non-uniform toughness and tissue elasticity. Therefore, it was decided to use the robot only to position and orientate the needle. Once the desired needle direction is reached by the robot, the surgeon's task is to insert the needle carefully. In this way, the experience of the clinician is taken into account who keeps in control of the surgery. Figure 2 shows the main system components in an interventional suite. The angiography system (Artis zeego, Siemens Healthcare) comprises a serial robot (KUKA Robots) with a C-arm attached to its wrist.

### 2.1. Workflow

Most robotic needle placement setups utilize the workflow introduced by Masamune *et al.* [25]. Although our system's workflow is similar, it has some different innovations. One of these is the so called target pivoting, which gives the flexibility to change the insertion point while the target is fixed. This method is not possible with RCM robots which normally use the workflow introduced by Masamune. The proposed workflow is described in the next steps:

1. **Preparation:** The patient is stabilized on the CT-table and a patient-image registration device is fixed according to the procedure described in [26].
2. **Imaging:** A 3D scan is acquired with the angiographic C-arm. The reconstructed CT-images are transferred instantly to the navigation suit.
3. **Planning:** Once the images are displayed on a touch-screen monitor, the clinician defines entry and target points.
4. **Interactive positioning:** The robot trolley is placed besides the patient. Then, the clinician takes the robot with the hand, activates the interactive positioning control (which will be described the next Section 2.3), and moves the robot arm till the tool tip is above the entry point.
5. **Automatic positioning:** Once the clinician activates the dead man switch the robot orientates the needle holder towards the planned target.
6. **Repositioning (teleoperation mode):** If required, the entry point can be changed using a joystick using the target pivoting option. During this procedure, the needle trajectory is continuously displayed in the 3D images.
7. **Needle insertion:** The needle is manually inserted by the clinician using the robot's needle holder as a guide. A confirmation scan can be performed with fluoroscopy or with a full-3D CT scan.
8. **Intervention or therapy:** Once the needle hits the target, the tissue sample can be taken or, in case of an ablation, therapy is performed.

### 2.2. Robot Control Modes

As shown in the workflow, the robotic system has three different control strategies:

***Interactive mode:*** The robot arm is freely maneuverable, as if it has no weight, in all directions of the Cartesian space (gravity compensation control). By picking the robot handle with the hand, and pressing sequentially two buttons attached to it, the interactive mode is activated. It is also possible to change the robot's elbow position by pushing it.

***Image guided mode:*** The robot moves according to patient-specific planning based data measured by the navigation system.

***Teleoperation mode:*** The user controls the robot arm with a joystick in TCP (tool center point) coordinates. Readjustments of the entering angle can be performed meanwhile the needle holder keeps pointing to the target. This is quite useful since the user can watch the new trajectory in the 3D images and choose the most convenient one.

### 2.3. Robot Trolley

**Figure 3.**
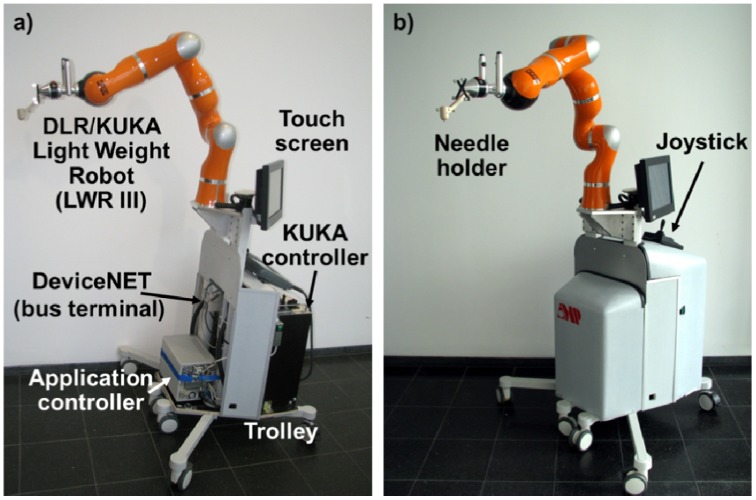
Mobile robot platform with mounted DLR/KUKA Light Weight Robot III. (**a**) The real-time robot controller, the application controller, a DeviceNET bus terminal and a touch screen are integrated; (**b**) Platform with covers attached to it.

As pointed out, the needle insertion robot is mounted on a mobile trolley, together with its manufacturer controller and a dedicated controller for the application (Figure 3). The trolley can be placed near the operating table so that the robot arm could be positioned near the patient without interfering with the C-arm trajectory. In order to do it intuitively, the robot has special control modes. The needle insertion robot is a third generation DLR/KUKA Light Weight Robot (LWR III) [7] especially designed for safe human-robot interaction. Due to the fact that this robot has carbon fiber covers and an aluminum skeleton it only weights 14 Kg. All sensors (including encoders, bumpers, and others), motor controllers and cables are integrated into the arm, which makes this robot good for manipulation in a crowded environment in which safety is of mayor concern. The robot has seven rotary joints; in contrast to six d.o.f. robots, its additional joint allows to change the elbows position without affecting the pose of the robot's tool. In every joint of the robot, a torque sensor measures the forces exerted. One of the mayor advantages of this setup is that the robot can be used in a so called gravy compensation mode. In this control mode, the robot arm can be moved by picking the robot with the hand with almost no resistance [27]. Once the user stops pulling or pushing it (on any part of its structure) it stays in its position waiting for the next movement. It looks quite similar like an object inside a spaceship, where gravity is not present. If some parameters of the control mode like virtual weight, friction and spring force (which can change the behavior of the compensation mode) are necessary to change, the programming interface of the manufacturer controller have the option to do it. At the bottom of the mobile platform are mounted the manufacturer real-time controller (KRC, KUKA Robot Controller) and the application controller. Attached to the trolleys inners frame there are different sensors for safety purposes which will be described in the control system section.

### 2.4. Grip and Needle Tool Holder

**Figure 4.**
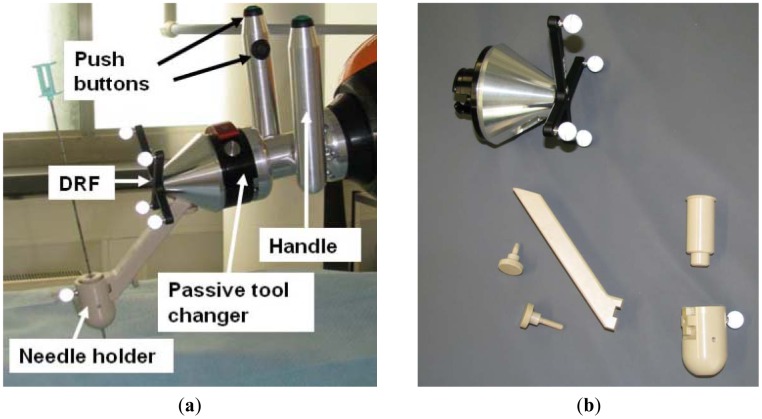
(**a**) robot handle together with the needle holder. A DRF is attached to the tool in order to be tracked by the navigation system; (**b**) the tool was designed autoclavable. The beige color parts are made of PEEK to ensure artifact-free imaging.

A handle with two grips is attached to the robot's wrist for user handling. The idea behind the use of two grips is that the user can take it from both sides of the patient table. Each grip has two push buttons, one at the top and the other at the inner side (Figure 4(a)). By pressing the push buttons, the gravity compensation mode can be activated. For safety reasons, this interactive mode can only be enabled when both push buttons are pressed, the upper one with the thumb and the lower one with the forefinger. At the end of the handle, a passive tool changer is mounted (GRIP GmbH Handhabungstechnik, Dortmund, Germany) in case the medical application needs a different tool. It can be seen in Figure 4(a) that, starting from the passive tool changer, the robot can be covered by a sterile drape to protect the robot from patient blood and other fluids.

In order to track the needle with the optical localizer, a dynamic reference frame (DRF) is attached to the needle holder (Figure 4(a)). The device that will carry the needle is the beige piece placed at the tool front. It is completely PEEK fabricated to ensure biocompatibility. In addition, its properties make this material artifact free in the CT-images. The needle holder can carry out different inserts to support varying needle or tool diameters.

### 2.5. Control System

As mentioned before, the KUKA/DLR LWR III is a serial robot. These kinds of robots have excellent repeatability but their absolute positioning accuracy is not outstanding due to small inaccuracies in their kinematics or calibration mistakes that increase over time. These inaccuracies have less impact when differential motions commands are given to the robot, meaning that the robot should move in relation to its last position instead of the absolute position. Based on this assumption, the present approach consists on locating the TCP position (Tool Center Point) and performing small movements taking the actual position as the origin.

#### 2.5.1. Application Controller

**Figure 5.**
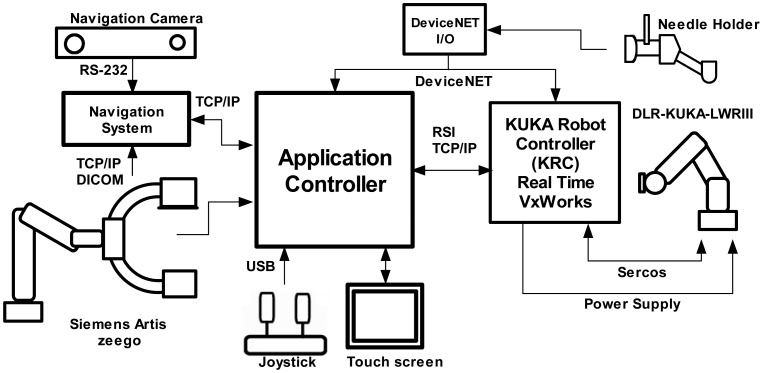
Control architecture. The application controller receives information from the different components of the system and uses it to control the robot arm.

The application controller main task is to centralize the data coming from all the system components, process this information and to send orders to be carried out by the actuators. The application controller gets sensor data from the real-time controller (robot pose and force measurements), the optical localizer (reference frames positions and orientations), the robot-driven angiographic system (2D projections and 3D-image reconstruction) a touch screen (user planning instructions) and a joystick (user command movements). The authors implemented the controller in a bare bone PC with Windows XP as operating system. Figure 5 shows an overview of the system components and their communication protocols with the application controller.

The application controller has a state machine which is triggered depending on the actual state and the data information coming from the system components. Examples of these transitions are when: the user introduces commands in the touch screen, an image is ready to use, the interactive control is activated, safety-related data from the KRC has arrived, *etc.* Robot internal safety-related features such as velocity limitation, force monitoring are processed in real time by the KCR. External safety-related emergency buttons and activating buttons (from the handle) are connected to the KCR and to the application controller though a DeviceNet link.

#### 2.5.2. Robot Sensor Interface

Dynamic data of the robot pose and movement's commands are cyclically exchanged through the KUKA Robot Sensor Interface (RSI) [28,29]. The RSI real-time interface is the solution offered by the robot manufacturer for coupling sensor to its controllers. Fundamental mechanisms of data communications are collected in the RSI, which is modularly structured and embedded into the KUKA programming environment. It supports synchronous and asynchronous data transportation based on industrial communication standards (Fieldbus, Ethernet). In this research, the data exchange between sensor (in this case the optical localizer) and robot is done using a XML messages. Sensor data is processed within the real-time kernel of the KUKA controller using predefined functions modules (*i.e.*, digital filters, transformations, control algorithms) that are combined in a sensor function library consisting of approximately 100 different modules. Processing tasks can be executed within one cycle time of Cartesian motion interpolation (12 ms) allowing that sensor signals can influence the robot positioning during motions.

A TCP/IP link from the KCR to the application controller was established in order to transfer XML data. In every interpolation cycle of the KRC an XML package containing the actual robot position, the joint angles, measured axis forces and motor currents are sent to the application controller. Based on this data, the application controller calculates an XML package including a correction vector for the TCP. The KRC processes the received package only if it arrives within the same time slot.

#### 2.5.3. Flow Diagram

**Figure 6.**
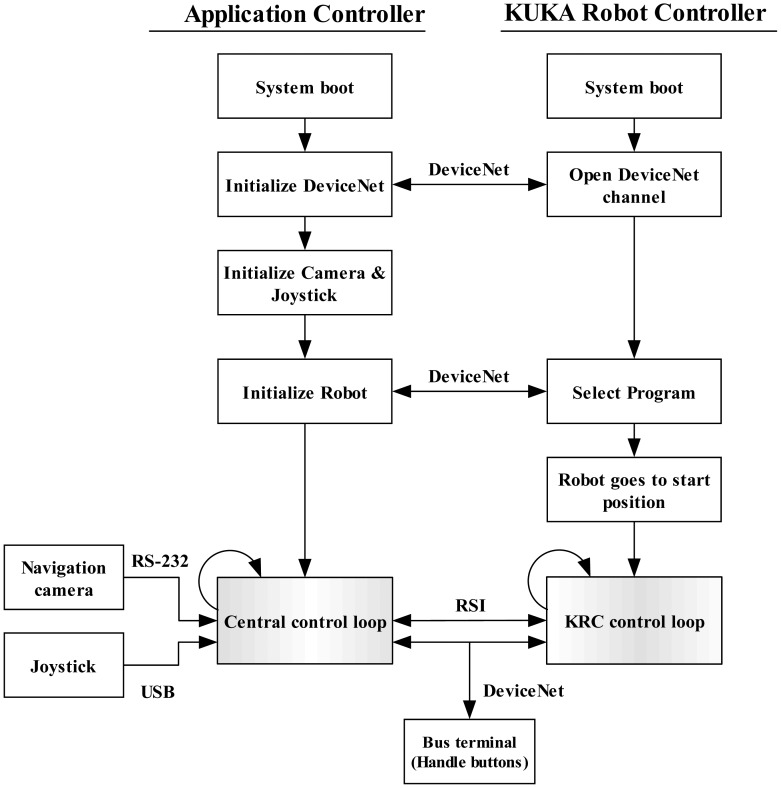
During initialization, all the channels necessary to make the application controller communicate with the main components of the system are opened. Then, the application controller and the KRC interchange information via RSI in order to control the robot motion.

The system program sequence can be seen in Figure 6. After booting, the application controller initializes the DeviceNet protocol and opens a communication channel with the KRC. Internal variables of the KRC that have direct influence on the robot's configuration and motion can be controlled externally by the application controller. Such variables can, for example, switch on/off the brakes, trigger an external stop, select a program, alert when a configuration lock is activated or the robot kinematic is not calibrated, *etc.* Then, the joystick and the camera are initialized. To do this, a USB channel is created for the joystick and a serial communication for the camera is established. Afterwards, the robot initialization is started. This includes program selection (on the KRC) using the opened DeviceNet channel. The security locks are then acknowledged and the robot brakes are released. Then, the robot moves to the programmed initial position. A TCP/IP channel is opened in order to exchange data through the RSI. After a command from the application controller, the RSI data exchange starts. This consists of two control loops, one in the application controller called central control loop and a second one in the KRC. Using the RSI, the control loops trigger each other every 12 ms. At every central control loop, the application controller receives data from the navigation system, the joystick and the bus terminal. Then, it sends the processed data to the KRC which in turn carries out the instructions (adjust the robot position). The KRC sends back a package containing the actual conditions of the robot. This data exchange is repeated until the user stops the program. If a delay exists, the data transmission is broken and the robot stops. The control loop is explained in more detail in the next section.

#### 2.5.4. Robot Control Loop

**Figure 7.**
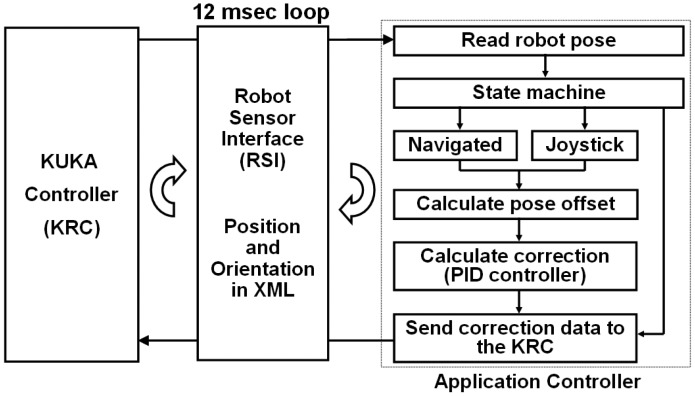
Control loop to manipulate the robot pose in the application controller.

The new position and orientation of the robot are continuously measured using the control loop in Figure 7. There are two ways to control the robot pose, either by the data obtained by the optical localizer or by the movement commands given by the user through the joystick.

The KRC transfers via RSI the actual robot's pose (of the wrist) in robot base coordinates. The state machine check which control mode is selected by the user. If the actual mode is “Navigation mode” the controller reads the tool and the patient anatomy positions and orientations and uses this information to calculate the TCP set point. Given the robot pose and the set point, the controller estimates the offset using some transformations that will be described in Section 2.6. In case the “Joystick mode” is activated then the measured value from this input device will be taken as an offset. Finally, in either case, a PID controller gets the correcting value that will be sent to the KRC in TCP coordinates.

#### 2.5.5. DeviceNet Link

A DeviceNet link is used to share additional inputs and outputs between the application controller and the KRC. Such I/Os work independently from the RSI communication and are used to initialize the KUKA controller, to share additional information and to be used as interruptions. When the robot handle buttons are pressed, this input is converted into a DeviceNet protocol by means of a bus terminal (BK5250, BECKHOFF New Automation Technology GmbH, Verl, Germany) and is finally shared with both controllers.

DeviceNet is a communication protocol used in the automation industry to interconnect control devices for data exchange. It uses a controller area network as the backbone technology and defines an application layer to cover a range of device profiles. Typical applications include information exchange, safety devices, and large I/O control networks. DeviceNet is a quite spread standard in the automation industry and is highly used by KUKA Robots to control their robots thanks to its real-time capabilities. KUKA controllers are equipped with a DeviceNet card that can be used to share information with external PLCs (programmable logic controllers) or computers.

**Figure 8.**
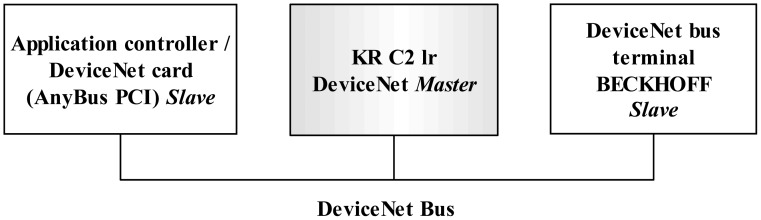
DeviceNet link. The data in the bus can be written/read by any of the connected cards.

The KR C2 lr controller was already equipped with a master DeviceNet link. Therefore a DeviceNet slave card was installed in the application controller to be connected with the one of the KR C2 lr. The card used is an AnyBus-PCI DeviceNet Slave with a baud rate of 500 Kbit/sec and 512 programmable I/O bytes (HMS Industrial Networks AB, Halmstad, Sweden). The DeviceNet data is shared between the application controller, the KUKA controller and the bus terminal (Figure 8).

**Figure 9.**
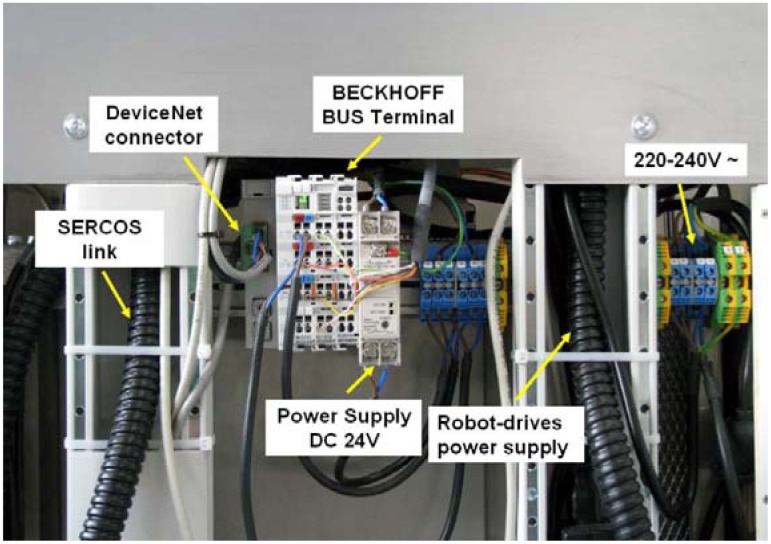
Inner connections of the robotic system, including a DeviceNet bus terminal and power supply.

The DeviceNet is mounted in the rack attached to the robot platform. Some of the physical connections can be seen in Figure 9, including the DeviceNet bus terminal, the power supply and the cables that control the robot system. The bus terminal uses a 24 Volt DC power supply which is distributed in a terminal.

### 2.6. Navigation

An optical localizer (Polaris, NDI, Waterloo, ON, Canada) was selected for the development and evaluation of the robotic system. In a final clinical setup, a commercial navigation system with a planning station will be used. The optical localizer tracks the position and orientation of dynamic reference frames (DRF). These frames have four retro-reflecting spheres which the optical localizer uses to detect with precision (with three or more spheres, it is possible to construct a coordinate system with its center in one of the spheres). The optical localizer update rate is 20 Hz and has a localization accuracy of 0.35 mm (rms). Since the control loop is running at 83 Hz an optical localizer with a higher acquisition rate would be a better option, but it was unfortunately not at hand. In this study, the optical localizer was not yet attached to the C-arm.

**Figure 10.**
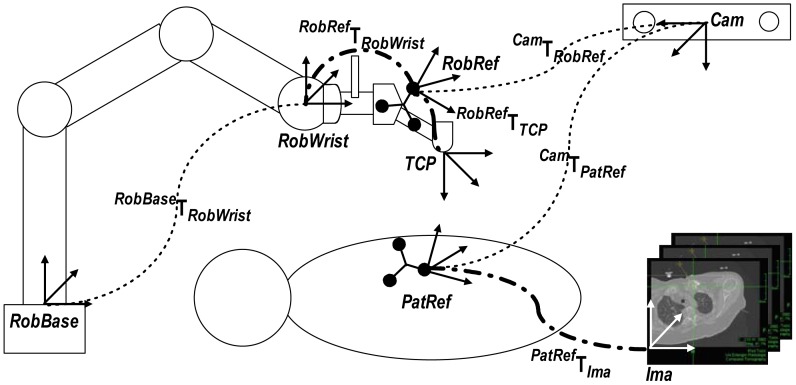
Coordinates and transformations of the system. The dotted lines represent the transformations measured by the navigation system and the robot controller. The dashed lines represent rigid transformations.

In Figure 10, all coordinate systems are presented. The optical localizer measures the transformations *^Cam^***T***_RobRef_* and the patient DRF *^Cam^***T***_PatRef_* in camera coordinates. Getting the transformation *^PatRef^***T***_Ima_* is the goal of the registration process.

The TCP is located at the lower end of the needle holder. During surgery, it has to be positioned above the skin surface pointing to the target in order to introduce the needle. The desired position of the TCP, *TCP_des_*, is selected in the CT data set (with *Ima* coordinates) in the planning step and can be, if necessary, changed with instructions coming from the joystick. The registration transformation *^PatRef^***T***_Ima_* is used to determine the transformation *TCP_des_* in relation to *PatRef*:

(1) TPatRefTCPdes=TPatRefIma⋅PImaTCPdes

The offset between *TCP* and *TCP_des_* can be calculated by:

(2) TPatRefTCP=TPatRefRobRef⋅PRobRefTCP

where

(3) TPatRefRobRef=[TCamPatRef]−1⋅PCamRobRef

and

(4) TTCPTCPdes=[TPatRefTCP]−1⋅PPatRefTCPdes

where *^TCP^***T***_TCPdes_* has be minimized to achieve the desired position.

**Figure 11.**
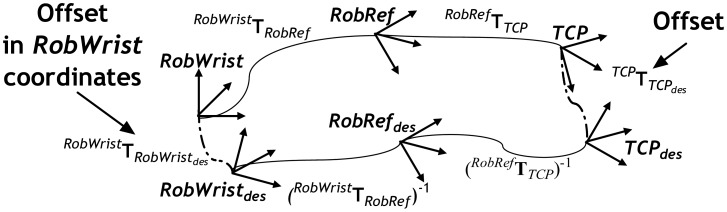
Offset transformation into RobWrist coordinates.

For robot control, the offset has to be transformed to *RobWrist* coordinates. To do this, the calibration transformation *^RobRef^***T***_RobWrist_* has to be first estimated (see Section 2.9). The chain of the last transformation can be seen in Figure 11.

### 2.7. System Transformations

In order to guide a needle into an anatomic area, the robot and the target area must be correlated. To do this, it is necessary to find a transformation between both coordinate systems. In the present approach, the optical localizer is used as an intermediate coordinate. The optical localizer tracks both the robot's actual position and the patient's actual position. The patient actual position measured with the optical localizer in relation to the 3D-image is carried out in the registration process. To correlate the robot with the optical localizer a calibration process is required.

### 2.8. Registration

In contrast to orthopedic procedures where the fixation of a DRF to bones is possible, needle placement procedures are performed on soft tissue. No rigid fixation is possible due to tissue deformation. Therefore, a special registration method introduced by Nagel *et al.*, was deployed [26]. The device shape utilized in this method reduces significantly errors introduced by tissue deformation. It consists of a DRF attached to a frame which has an empty space (to insert the needle) in the center and CT-markers distributed in known geometry. A vacuum bag is used to stabilize patient movements. The transformation from the DRF to the CT-markers coordinate system is known in advance and is used to get the transformation *^PatRef^* **T***_Ima_* that registers the patient image to the navigation system.

### 2.9. Calibration

The calibration process consists in finding the transformation *^RobRef^*T*_RobWrist_* and the transformation *^RobRef^*T*_TCP_*. Once these transformations are estimated the whole chain of the system transformations are known. For the user, the calibration procedure consists of two simple steps:

1. Robot pivoting. A small iron tip is inserted into the needle holder. The robot is taken using the gravity compensation mode and the iron tip is inserted into a fixed divot. With the optical localizer pointing to the robots tool, the user pivots the robot doing smooth rotational movements for about 30 seconds.
2. Automatic sequence. A reference DRF is attached near the robots base (always within the camera volume of measurement). After a user command, the robot follows a sequence of movements.

Internally, during the pivoting step, *^RobWrist^***P***_TCP_* and *^RobRef^***P***_TCP_* are estimated. Both are necessary to calculate *^RobRef^***T***_RobWrist_* as will be described later. In the automatic sequence an algorithm to get *^RobRef^***T***_RobWrist_* is executed.

The calibration method implemented in this research is similar to the used in medical robotics applications with similar setups [24,30]. This method consists in finding the transformation from a fixed DRF (*Ref*) to the robot base coordinate system *RobBase*, *^Ref^***T***_RobBase_*, which is only useful for calibration. With this matrix, the transformation *^RobRef^***T***_RobWrist_* is then estimated. Robot pivoting helps to find *^RobRef^*P*_TCP_* and *^RobWrist^*P*_TCP_*. In step two, the robot TCP is moved through a particular workspace in order to measure two sets of corresponding TCP points in relation to the camera and the robot base. By matching these two datasets the transformation *^Ref^***T***_RobBase_* is calculated with a pair point method.

#### 2.9.1. Robot Pivot Calibration

The pivot calibration consist on the repetitive tilting (pivoting) of a rigid instrument tip into a small orifice called divot along two spherical axes [31]. The gravity compensation mode described in Section 2.3 was used for this purpose. Once this mode is activated, the robot handle was taken with the hand and inserted into the divot of a fixed aluminum plate. While pivoting, the transformation *^RobBase^***T***_RobWrist_* was continued obtained from the KRC and saved in a text file. At the same time, the transformation *^Cam^***T***_RobRef_* was also obtained and saved in a text file. The *TCP* translations relative to coordinate systems *RobBase* and *Cam* are estimated by finding the most invariant point in these pivot motions as:

(5) [RRobBase(1)RobWrist|⋮⋮|−IRRobBase(n)RobWrist|⋮][PRobWristTCP−PRobBaseTCP]=[P_RobBase(1)RobWrist⋮P_RobBase(n)RobWrist]

(6) [RCam(1)RobRef|⋮⋮|−IRCam(n)RobRef|⋮][PRobRefTCP−PCamTCP]=[P_Cam(1)RobRef⋮P_Cam(n)RobRef]

where *n* is the number of measurements, *^RobBase^*R(i)*_RobWrist_* the rotation matrix, *^RobBase^*P(i)*_RobWrist_* the position vector of a single measured pose of the robot wrist in robot's base coordinate system. *^Cam^*R(i)*_RobRef_* and *^Cam^*P(i)*_RobRef_* built the pose of the attached DRF in camera coordinates; –I is the identity matrix. To find the desired values Equations (5,6) can be expressed as:

(7) [PRobWristTCPPRobBaseTCP]=[ℝRobWristRobWristT⋅ℝRobBaseRobWrist]−1⋅ℝRobBaseRobWristT⋅ℙRobBaseRobWrist

(8) [PRobRefTCPPCamTCP]=[ℝCamRobRefT⋅ℝCamRobRef]−1⋅ℝCamRobRefT⋅ℙCamRobRef

*^RobBase^*ℙ*_RobWrist_^RobBase^*ℝ*_RobWrist_*, *^cam^*ℙ*_RobRef_* and *^Cam^*ℝ*_RobRef_* represent the complete set of measured poses during pivoting. Since Equations (7,8) are not square matrices, the unknowns are solved in the least squares sense. Note that in pivot calibration, the final result contains only the translation vector.

The pivot process took about 1 minute and n = 1,000 different poses were obtained. The maximum pivot angulation was of 120°. To estimate the quality of the procedure, the residual error was computed as:

(9) erms(PRobWristTCP)=1N[ℝRobBaseRobWrist[PRobWristTCPPRobBaseTCP]−ℙRobBaseRobWrist]2

(10) erms(PRobRefTCP)=1N[ℝCamRobRef[PRobRefTCPPCamTCP]−ℙCamRobRef]2

The result for the navigation part is typical for pivot calibration (see Table 1). The higher error value of the robot calibration may be attributed to mechanical instabilities. However, the error influence is not as big in the final calibration result due to the close loop control strategy.

#### 2.9.2. Pair Point Method

**Figure 12.**
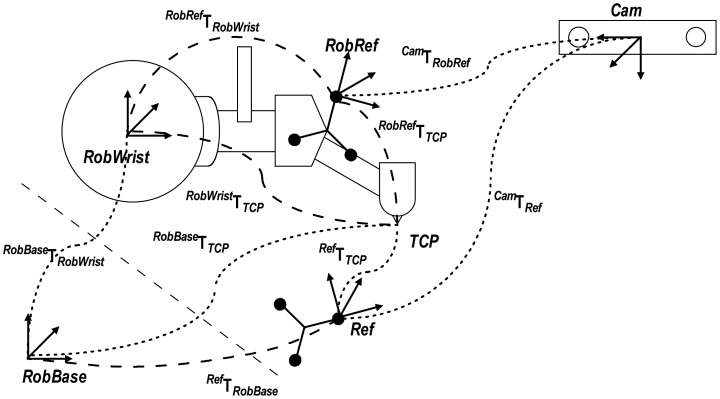
To obtain the rigid transformation *^RobRef^*T*_RobWrist_* first the position of the TCP is measured using a pivot calibration following by a pair-point method with an additional DRF (*Ref*).

With the translation vectors, derived from the pivot procedure, the transformation *^Ref^*T*_RobBase_* can be calculated using the pair point method (Figure 12). This method consists of estimating the transformation between two coordinate systems by using singular value decompositions of a covariance matrix of a set of corresponding points [32]. For both sets of measured points ${\left\{{\text{a}}_{i}\right\}}_{i=1}^{n}$ and ${\left\{{\text{b}}_{i}\right\}}_{i=1}^{n}$ the relation can be described by:

(11) $$b_{i}\approx R\cdot a_{i}+P,i=1,2,\ldots ,n,a_{i},b_{i}\in {ℜ}^{3}$$

The similarity transformation parameters (R: rotation, P: translation) give the minimum value of the mean squared error *e*^2^(R,P) of these two point sets:

(12) $$e^{2}(R,P)=\frac{1}{n}\sum_{i=1}^{n}{\left\|b_{i}-(Ra_{i}+P)\right\|}^{2}$$

For a detailed description of the used least square fitting algorithm method, utilized to minimize *e*, the reader may refer to the work by Umeyama [32].

Selecting the TCP coordinate system as a mutual point to match both measurement systems (the robot encoders at one side and the navigation camera on the other) it is possible to calculate the rigid transformation between them. The unknown transformation matrix *^Ref^***T***_RobBase_* can be determined once by moving the robot along the work space and calculate the TCP position using Equations (7,8):

(13) PRobBaseTCP=TRobBaseRobWrist⋅PRobWristTCP

(14) PRefTCP=[TCamRef]−1⋅TCamRobRef⋅PRobRefTCP

#### 2.9.3. Determination of the Rigid Transformation

The robot was moved to 175 different programmed positions. At every position the robot was stopped for 5 seconds. To filter noise, 100 measurements were taken at every position; the calculated mean values were then used in the following procedure. 50 positions were distributed equally in the robot's workspace; these measured points were used to determine *^Ref^***T***_RobBase_* according to the method described before.

The leftover 125 measured points were used to estimate the residual error of the obtained *^Ref^***T***_RobBase_* matrix and were distributed equally within a work space of (200 mm)^3^. Two different transformation chains were used to calculate the resulting difference at each point, called estimated error:

(15) TRefTCP(short)=TRefRobRef⋅TRobRefTCP

(16) TRefTCP(long)=TRefRobBase⋅TRobBaseRobWrist⋅TRobWristTCP

In an ideal case the difference between these two equations should be zero:

(17) [TRefTCP(short)]−1⋅TRefTCP(long)≈I

By applying Equation (17) to the 125 different measurements a root mean square residual error of the length of the translational part was 0.81 mm with a standard deviation of 0.41 mm (Table 2).

As described before, the system's control loop uses *^RobRef^***T***_RobWrist_*, this can now be determined by calculating:

(18) TRobRef(i)RobWrist=[TRef(i)RobRef]−1⋅TRef(i)RobBase⋅TRobBase(i)RobWrist,i=1…n

for all measured positions. The noise is filtered by using the mean values for every field of the resulting matrix. The matrix was made homogeneous afterwards.

### 2.10. User Control Modes

**Figure 13.**
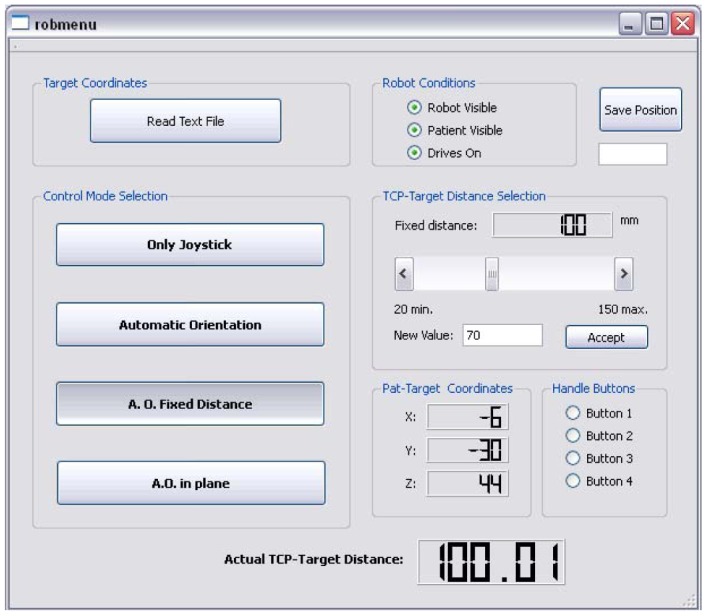
Graphical user interface. The user may select the desired control mode and trajectory coordinates. Also, the interface shows whether there is visual contact between the robot and the patient′s camera.

A graphical interface (GUI) was designed to easily select the functions of the robot (Figure 13). The target coordinates in relation to the patient can also be read from a text file. It also tells the user when the robot and the patient are not visible to the navigation camera. The actual distance from the TCP to the target is continuously displayed.

**Figure 14.**
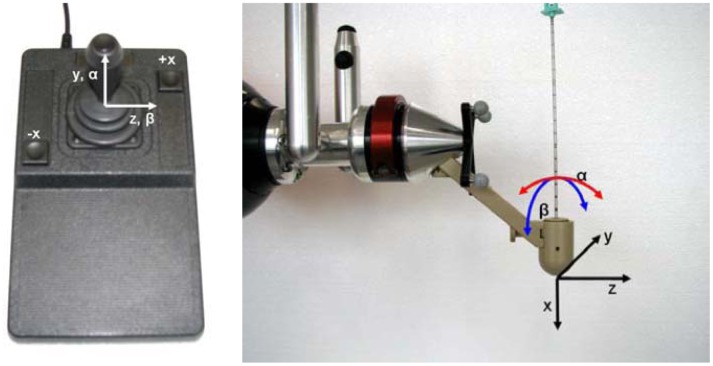
An industrial joystick is used to move the TCP in Cartesian coordinates. By pressing the joystick's upper button pivoting at the TCP is possible.

The user control modes are explained next:

***Only joystick***. In this control mode the TCP can be moved in Cartesian coordinates according to Figure 14. A joystick movement to the left corresponds to a robot movement to the left and so on for right, front and back movements. By pressing the side joystick buttons the robot can be moved back and forth along the needle direction (x direction in TCP coordinates) at constant velocity. The robot calculates internally all the necessary transformations. If the user wants to change the TCP orientation (but keep the position) he only has to press the upper button of the joystick to initiate movement along α and β (see Figure 14).

**Figure 15.**
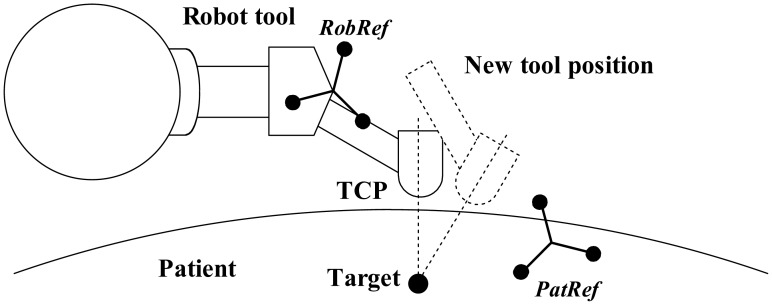
In automatic orientation mode the robot orients the TCP in relation to the target every time after a joystick movement is performed. If the patient moves, the robot will react and compensate for patient movement and will point again to the target.

***Automatic orientation***. Once this mode is selected, the robot orients the TCP automatically towards the target as shown in Figure 15. It is still possible to change the TCP position using the joystick like in “only joystick mode” but once the joystick is released (a new desired position is achieved) the robot orients itself again pointing to the target but now from a new perspective. This control mode is quite helpful to find new entry points. During the operation, the radiologist can move to different entry points and decide which one may be more adequate. This control mode may be more helpful to radiologists than the mentioned RCM method where only one entry point can be chosen (otherwise the whole Cartesian positioning has to be repeated, which involves moving the robot manually to a different location at the skin and then try to pivot again).

**Automatic orientation with fixed distance:** This control mode works similarly to automatic orientation mode with the only difference that the user can decide on the trajectory distance. The desired distance can be entered by the user on the GUI.

**Automatic orientation in plane:** By selection of this mode the TCP can be moved with the joystick only along an imaginary plane positioned over the patient skin. The TCP keeps pointing at the target at any time.

## 3. Results and Discussion

Preliminary accuracy tests of the developed components and procedures were performed. The overall chain error includes errors introduced by the imaging system, planning, patient registration and unrecognized movement of patient tissue. Additional errors are introduced by the robotic system and its connection to the navigation system, namely robot kinematic error, robot calibration error, navigation system measurement error and instrument calibration error. These errors were evaluated in three experiments. The first two experiments, namely the evaluation of the kinematics and the imaging system error can be seen in [10] which show that the robot is able to reach positioning with accuracy similar to the optical localizer, 0.35 mm (rms). In this article, only the overall error was measured in the next experiment.

### 3.1. Accuracy Tests for Targeting a Needle

**Figure 16.**
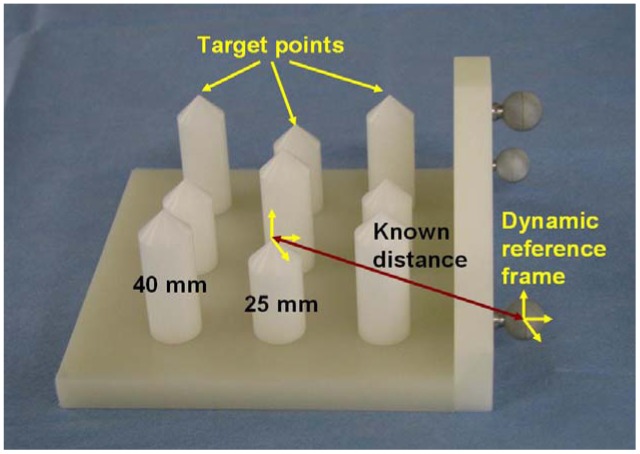
Accuracy testing device. The rod's tip position in relation to the attached DRF was known in advance.

These measurements were performed using a specially designed testing device and an Artis zeego imaging system for error visualization. The testing device consists of nine rods with tips distributed along different positions Figure 16. The height of the higher five rods was 40 mm (from the base to the tip) while the height of the smaller four rods was of 25 mm. A DRF was attached to one side of the testing device. The distances from the DRF's coordinate system to the rod tips were known in advance. The construction accuracy of the testing device is about 0.01 mm.

**Figure 17.**
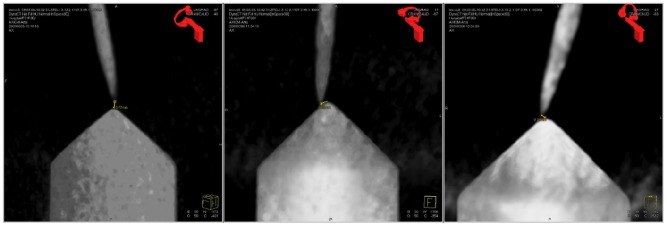
The error was defined as the distance between the needle's tip and the rod's tip measured on the reconstructed CT-images.

For the experiment, the trolley with the robotic system was placed at one side of the CT-table. The robot's tool was positioned over the testing device, which was positioned on the CT table, using the gravity compensation mode. Using the graphical interface, the robot system was programmed with the position of the selected tip. The TCP was positioned accordingly over the selected tip using the joystick control mode. After an automatic orientation command, selected in the graphical interface, the robot orientated the TCP pointing to the target. The angle from the rod's vertical to the TCP did not exceed 45 degrees. The experiment was performed using different trajectories lengths at each rod (30–60 mm). Then, the robot was stopped by activating the robot's brakes and a 150 mm needle with 2 mm of diameter was inserted until the tip reached the rod's peak. A CT scan (20 s, 200° rotation range) was performed and reconstructed using a high resolution kernel with 512 × 512 matrix and 0.13 mm voxel size. The distance error was defined as the measured distance from the needle's tip to the rod's tip in the CT images (Figure 17).

The experiment was repeated approaching from five different directions for all rods, resulting in a total of 45 measurements. The resulting root mean square positioning error e_rms_ is shown in Table 3 together with its standard deviation σ and the minimum and maximum deviation e_min_ and e_max_ respectively.

## 4. Conclusions/Outlook

In this research a fully sensorized cooperative robot system for surgical interventions was introduced. These kinds of systems depend heavily in the information coming from different sensors. Therefore, in this paper the primary focus lies in the sensor technology employed. The robot system was adapted for the placement of needles into anatomic areas such as liver, kidneys, and lungs. In these kinds of operations, an interventional radiology procedure is commonly required for target visualization. The system uses an optical localizer for robot control and patient tracking. For target visualization, a robot-driven FD-CT was introduced, which gives the systems the flexibility to move along the patient table with the use of joysticks and pedals. The developed mobile robot platform can be easily positioned in an intraoperative suit. The LWRIII robot control strategies allow robot manipulation with the hand. For fine movements, the robot can be manipulated via joystick while the target is fixed, helping the clinician to choose different entry points. The autoclavable tool holder can support different kinds of tools for different robot operations. An application controller was developed specially for surgical applications which require real time response. Real time control was possible due to the RSI-Ethernet.

Because of the existence of the different system components, namely the robot arm, the robot-driven FD-CT and the optical localizer, a calibration process was required. For the clinician, this calibration process is easy to carry out without the need of a technical assistance. Once calibration was done, it was visually confirmed that the robot reacted faster to new programmed poses. No meaningful oscillations were presented in the steady state. When the patient reference frame was manually moved with slow movements, the robot mirrored the movement smoothly. Nevertheless, for big movements the robot does not reacts fast enough to mirror the movements. Therefore, it can be claimed that the proposed setup is able to compensate only for small patient movements. Using Kalman filters and using an optical localizer with faster frequency (100 Hz) would improve this reaction.

While targeting points with the robot system the whole error chain is present. The most significant error includes the robot calibration error, the optical localizer error, the testing device construction error, and the image reconstruction error. The obtained error of 1.2 mm with a standard deviation of ±0.4 mm seems to be acceptable but insufficient for some critical applications. Using navigation camera with a higher accuracy and smaller robot like the MIRO [33] may improve the accuracy.

With the used components, a line of sight problem emerges. This is mainly due to fact that many components in the same working space are present. In this research, we partially solved this problem by attaching the navigation camera to the C-arm as shown in Figure 1. Moving the C-arm will not affect the camera measurements as they are done in relation to a DRF. Finally, the navigation data could also be used for real-time 3D reconstruction.

## Figures and Tables

**Table 1.** Pivot residual error (n = 1,000).

**Rigid transformation**	***e****_rms_*
*^RobWrist^***P***_TCP_*	0.94 mm
*^RobRef^***P***_TCP_*	0.47 m

**Table 2.** Residual error.

***e****_rms_*	**Standard Deviation**
0.81 mm	0.41 mm

**Table 3.** Technical accuracy results for targeting a needle on the accuracy testing device. The accuracy was determined as the distance from the needle's tip to the rod's tip from N = 45 different measurements.

**e_rms_ ± σ**	**e_min_**	**e_max_**
1.2 mm ± 0.4 mm	0.33 mm	1.98 mm
